# Triple-induction treatment for locally advanced non-small cell lung cancer: a case report of pathological complete response

**DOI:** 10.1186/s13019-024-02759-y

**Published:** 2024-04-15

**Authors:** Raphael S. Werner, Olivia Lauk, Georg Tscherry, Alessandra Curioni-Fontecedro, Sylvia Höller, Isabelle Opitz

**Affiliations:** 1 Department of Thoracic Surgery, University Hospital Zurich, Rämistrasse 100, Zurich, 8091 Switzerland; 2 Department of Oncology, Regional Hospital Uster, Brunnenstrasse 42, Uster, 8610 Switzerland; 3 Department of Medical Oncology and Hematology, University Hospital Zurich, Rämistrasse 100, Zurich, 8091 Switzerland; 4 Present address: Clinic of Oncology, Cantonal Hospital Fribourg, Fribourg, 1752 Switzerland; 5 Faculty of Science and Medicine, University of Fribourg, Fribourg, Switzerland; 6 Department of Pathology, University Hospital Zurich, Rämistrasse 100, Zurich, 8091 Switzerland; 7 Present address: Institute of Clinical Pathology, Stadtspital Zurich, Birmensdorferstrasse 497, Zurich, 8063 Switzerland

**Keywords:** Advanced lung cancer, Pneumonectomy, Multimodality treatment, Pathological complete response, Case report

## Abstract

**Background:**

In patients with resectable stage III non-small cell lung cancer (NSCLC), induction chemoimmunotherapy followed by surgical resection has shown unprecedented rates of pathological response and event-free survival. However, a triple-induction including radiochemotherapy and immunotherapy followed by surgical resection has not been routinely established in clinical practice.

**Case presentation:**

We report the case of a 47-year-old patient with stage IIIA NSCLC who was treated in a combined concept including induction concurrent radiochemotherapy, followed by 4 cycles of pembrolizumab and subsequent intrapericardial left-sided pneumonectomy. Histological analysis revealed a pathological complete response.

**Conclusions:**

The case demonstrates that the combination of neoadjuvant chemo-, radio- and immunotherapy in advanced NSCLC may lead to a relevant down-staging and may enable a R0-resection of a borderline resectable tumor. However, the combination of four different treatment modalities requires resilience and a good performance status. A triple induction treatment may be a promising option for selected patients with locally advanced NSCLC and good performance status.

## Background

Among patients with non-small cell lung cancer (NSCLC), approximately one third presents with stage III at initial diagnosis [1]. The treatment of patients at this stage remains challenging due to the local extension of the primary tumor and the increased risk of metastatic progress. It is not surprising, that with the increased risk of local or distant recurrence in this stage, the historically reported 5-year survival in resected stage III NSCLC is below 36% despite conventional chemotherapy [2]. While neoadjuvant chemoimmunotherapy has become the preferred treatment of resectable stage III NSCLC in many countries, the addition of neoadjuvant radiotherapy is not commonly used in clinical practice.

Here, we report the case of a patient with stage IIIA NSCLC who was treated in a quadrimodal concept by combining the neoadjuvant chemoradiotherapy with immunotherapy and subsequent surgery.

## Case presentation

**Fig. 1:**
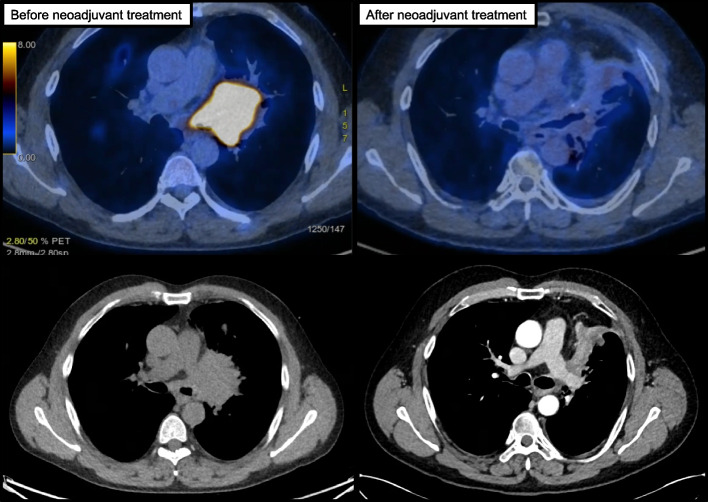
Positron-emission computed tomography (PET-CT) and computed tomography (CT) before initiation of neoadjuvant treatment (left) and after administration of neoadjuvant chemoradiotherapy and immunotherapy (right). The contrast-enhanced CT (left, below) depicts the left main pulmonary artery encircled by the residual tumor mass

A 47-year-old male patient with a smoking history of 70 pack years presented with shortness of breath and minor hemoptysis. A computed tomography (CT) of the chest revealed a large tumor of the left hilum with infiltration into the upper- and lower main bronchus, infiltration into the left pulmonary artery and subcarinal extension. Upon subsequent bronchoscopy and transbronchial biopsy, a squamous cell carcinoma was diagnosed. PD-L1 immunohistochemistry (clone E1L3N®, Cell Signaling Technology, Cambridge, UK) revealed a positivity in 40% of the tumor cells and in 70% of the tumor-infiltrating immune cells. A staging by positron emission computed tomography (PET-CT) showed normal-sized mediastinal lymph nodes without increased FDG-uptake, but metabolically active left axillary lymph nodes. The axillary lymph nodes were thus resected and histological analysis showed focal lymphadenitis, but no malignancy. After a cranial magnetic resonance tomography had displayed no signs of brain metastases, a clinical T4 N0 M0 status was defined, corresponding to UICC stage IIIA. Therefore, an induction concurrent radiochemotherapy was initiated. Induction chemotherapy consisted of three cycles of carboplatin and paclitaxel. The concurrent neoadjuvant hyperfractionated radiotherapy was administered to a cumulative dose of 50 Gy in 25 fractions of 2 Gy, given 5 days a week. The subsequent CT showed a partial morphological response. Considering the high PD-L1 expression, the patient subsequently received 4 cycles of pembrolizumab. The re-staging by PET-CT (Fig. 1) revealed a further reduction in tumor size with only minimal residual metabolic activity and no relevant new lesions.

**Fig. 2:**
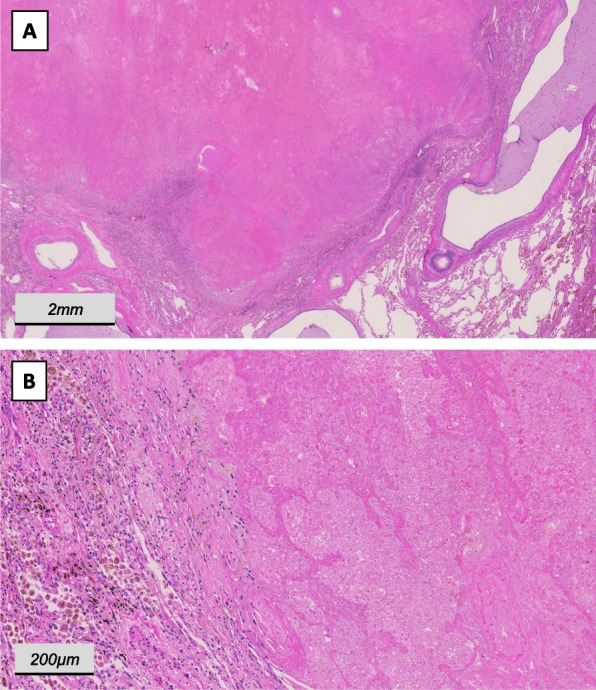
Image **A** shows an overview of the former tumor bed composed of necrotic tissue in the center (top left) surrounded by a rim of sparse fibrosis and a more prominent inflammatory infiltrate. At higher magnification (**B**), isolated structures such as indicated vessels and completely necrotic tumor nests can be seen within the necrosis zone (right). The adjacent transition zone between necrosis and normal lung tissue shows a high proportion of partially haemosiderin-laden macrophages and scattered lymphocytes

The patient was then admitted for surgical resection as definitive local treatment 5 weeks after the last cycle of immunotherapy. Pre-operative spirometry showed sufficient functional reserve (forced expiratory volume in one second: 2.75L (75% of set), diffusing capacity 64% of set) and the perfusion of the diseased left lung (V/Q-scan) was reduced to 29%. Due to the central location of the tumor with contact to the left main pulmonary artery and bronchus, an intrapericardial left-sided pneumonectomy was performed, followed by mediastinal lymphadenectomy. Upon histological analysis, a pathological complete response (pCR) was found, correspondent to a ypT0 ypN0 (0/25) status (Fig. 2). The patient was discharged after an uneventful recovery on the 11^th^ postoperative day. Postoperatively, the patient reported a mildly to moderately impaired respiratory reserve upon physical exertion. In the last regular clinical and radiological follow-up 24 months after surgery, no signs of relapse were present.

## Discussion and conclusions

Our case demonstrates that the combination of radiochemotherapy and immunotherapy in a neoadjuvant setting of a patient with stage IIIA NSCLC may result in a pCR and enable an R0-resection of an otherwise unresectable tumor. In the past years, the use of immune checkpoint inhibitors that act by restoring the antitumor immune response have shaped the treatment landscape in locally advanced NSCLC with unprecedented response rates. Especially in the neoadjuvant setting, immunotherapy may enhance the immune response by using the primary tumor bulk as an antigen source for activation of cancer-specific T-cells [3]. Similarly, after cancer irradiation an increased systemic anti-tumor immune response is seen by the so-called abscopal effect that defines a radiotherapy-induced release of tumor neoantigens and modulation of the tumor immune microenvironment [4]. Both neoadjuvant chemoimmunotherapy and neoadjuvant radiotherapy not only help to down-stage the tumor before surgery, but also aim to treat micrometastatic disease by generating a systemic antitumor response [3, 4]. The benefit of neoadjuvant or perioperative chemoimmunotherapy in resectable stage II to IIIB patients has recently been demonstrated by several phase III trials: in the treatment arms, the Checkmate 816 trial [3], Checkmate 77t trial [5], Keynote-671 trial [6], AEGEAN trial [7] and Neotorch trial [8] report exceptional pCR rates of 24%, 25.3%, 18.1%, 17.2% and 24.8%, respectively and event-free survival rates of 62.4% to 73.2% at 2 years. While definitive radiochemotherapy followed by immunotherapy is regularly used in the clinical practice for treatment of locally advanced, unresectable NSCLC according to the PACIFIC trial’s protocol [9], the combination of these treatment modalities in the neoadjuvant setting of resectable disease is not routinely used in clinical practice. Molecular analyses of the tumor microenvironment before and after chemo- or radiotherapy have demonstrated that induction strategies may create a more immunogenic microenvironment, including higher densities of helper T-cells and tumor-associated macrophages, as well as an increased infiltration of CD8 + cytotoxic T-cells [10]. By priming a non-inflamed (“cold”) tumor, the efficacy of a subsequent immune checkpoint inhibition may therefore be improved. This effect is clinically shown by the sustained and durable 5-year survival benefit reported in the PACIFIC trial with a 5-year OS of 42.9% in the group receiving durvalumab as consolidation treatment in unresectable stage III NSCLC [9]. The effect of an immune-modulatory radiotherapy for an enhanced effect of neoadjuvant immune checkpoint inhibition after neoadjuvant chemotherapy for stage III (N2) NSCLC is currently investigated in the SAKK 16/18 trial [11].

In our reported case, the treatment response and the patient’s performance status directed the multidisciplinary tumor board to recommend a surgical resection within a salvage approach. The excellent pathological response however raises the question in retrospect, whether surgical resection was indeed needed. Pathological response currently holds the highest predictive value for event-free survival when compared to the radiographic response or circulating tumor DNA clearance, and may ultimately guide subsequent adjuvant therapy in the perioperative setting [12]. A biopsy-guided pathological response assessment has been recently investigated in other cancer entities and is an insufficient predictor of pCR [13, 14]. In our case, the good functional reserve and the reduced perfusion of the left lung in the V/Q scan indicated that a left pneumonectomy was safe and feasible. Nevertheless, these decisions always need to be made by a multidisciplinary tumor board, taking the patient’s performance status, as well as other local treatment approaches into consideration.

In consideration of the excellent pathological response in our described case, the triple induction treatment may be a promising option for selected patients with locally advanced NSCLC and good performance status. Further research is required to identify pathological response before surgery.

## Data Availability

Not applicable.

## Abbreviations

CT: Computed tomography
NSCLC: Non-small cell lung cancer
OS: Overall survival
pCR: Pathological complete response
PFS: Progression-free survival
PET-CT: Positron emission computed tomography
